# Miniaturization of frequency selective surface by 2.5-dimensional meandered split ring cells for application in L-band

**DOI:** 10.1038/s41598-023-46159-z

**Published:** 2023-10-31

**Authors:** Amir Khajevandi, Homayoon Oraizi

**Affiliations:** School of Electrical Engineering, Iran University of Science and Technology, Tehran, 1684613114 Iran

**Keywords:** Engineering, Electrical and electronic engineering

## Abstract

In this research article, A miniaturized hexagonal split-ring 2.5-dimensional (2.5-D) unit cell is proposed for frequency selective surface (FSS) applications. The 2.5-D FSS provides efficient usage of the available surfaces and optimum increased current paths by connecting its two sides through vias. The size of the FSS unit cell is $0.026{{\varvec{\uplambda}}}_{0}\times 0.026{{\varvec{\uplambda}}}_{0}$ at the resonance frequency of 1.25 GHz. The proposed FSS has good angle stability due to its small size. The effects of variation of FSS geometry parameters on transmission zero are investigated, namely, the control of bandwidth and roll-off of frequency response by these parameters is studied. An equivalent circuit is obtained for the proposed structure to predict its frequency response, which agrees very well with the results of full-wave simulations. A prototype model of the proposed FSS structure is designed, simulated, fabricated, and tested as proof of concept.

## Introduction

Frequency selective surfaces (FSSs) are designed as periodic arrays that respond differently to electromagnetic waves at different frequencies and operate as spatial filters, which have wide applications in communication systems. Various responses are realized by different unit cell element shapes and geometries, such as rings, square patches, fractal slots, or patches. Different structures and geometrical configurations have been proposed for FSSs. They often display selectivity on the angle and polarization of the incident wave^1^. Recently, FSSs have been extensively used in microwave applications, such as design of radomes^2^, shielding^3^, and improvement of antenna characteristics^4^. Due to their extensive applications at various frequencies, their investigations have been popular in microwave and optical problems for decades, such as the communication capabilities of satellite platforms. FSSs are commonly fabricated as two-dimensional (2-D) periodic arrays on dielectric substrates, whereby their frequency selective properties and space filtering characteristics are employed.

Due to space limitations in practice, it is important to employ an FSS structure with an infinite number of unit cells. Consequently, it is necessary to obtain the response of an infinitely large FSS by a finite number of unit cells. Therefore, the miniaturization of FSS unit cells is a required task in practice^1^. In the process of FSS miniaturization, the angular stability should also be realized^5^, but at high angles of incidence, the resonance frequencies usually change, due to the unit cell geometry. One of the methods implemented for the broad banding of FSSs is the application of fractal geometries for the unit cell, which also brings about miniaturization^6,7^. Recently, a new miniaturized type of FSS unit cells called MEFSS has been introduced in the literature^8^, a variety of structures have been introduced for the miniaturization of FSS, such as using lumped elements^9^, interdigital capacitance structures^10^, closed metallic loops and their complementary loops^11^ and 2.5-D close loop FSS^12,13^.

In this paper, a miniaturized hexagonal stop band unit cell FSS with the incident angular stability, and polarization dependence characteristics is proposed which is designed by the 2.5-D meandered split ring in the L-band. The proposed FSS is built on a substrate with two metalized surfaces which are connected by vias, and it has very good angular stability for the incident angle of 0° to 60° which achieves a miniaturized size of $0.026{{\varvec{\uplambda}}}_{0}\times 0.026{{\varvec{\uplambda}}}_{0}$.

### Miniaturization of a hexagonal unit cell

**Figure 1:**
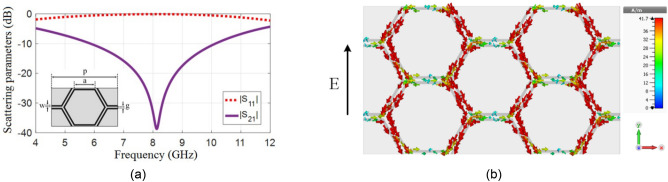
Hexagonal unit cell; (**a**) Transmission and reflection responses. (**b**) Current density at frequency 8.08 GHz.

Figure 1a shows the transmission and reflection coefficients of the proposed hexagonal cell for the TE-mode wave incidence. The current density at the resonance frequency (≈ 8.06 GHz) is shown in Fig. 1b, where a current density with one wavelength long appears on the cell.

**Figure 2:**
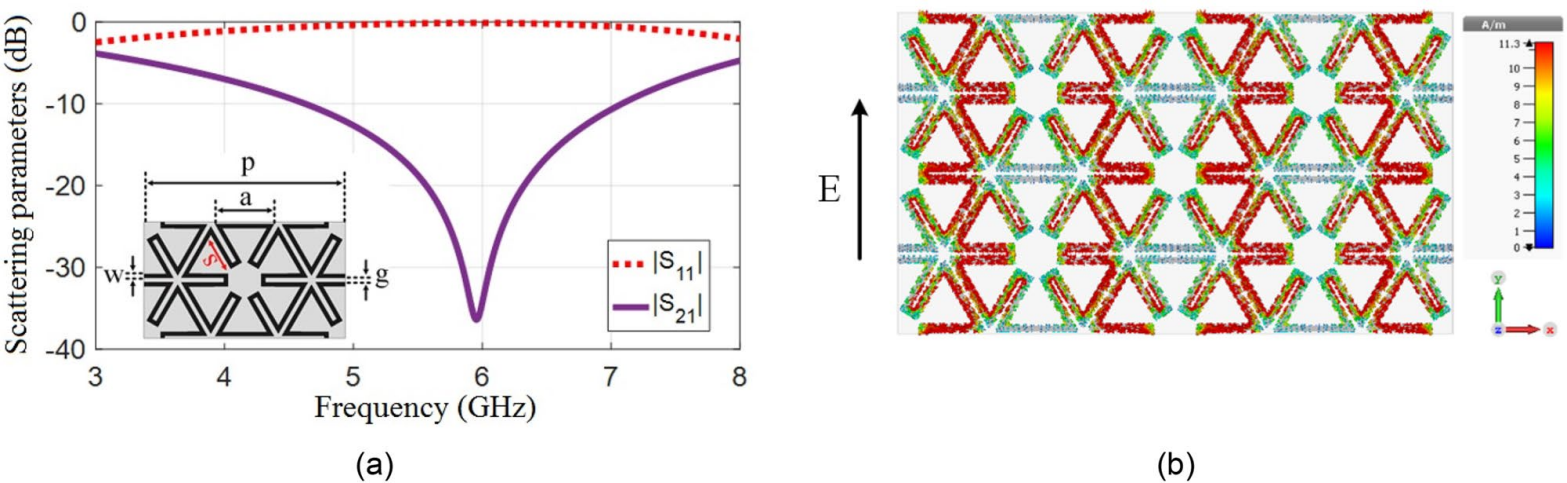
Hexagonal meandered unit cell, (**a**) Transmission and reflection responses, (**b**) Current density at 5.9 GHz.

The unit cell may be effectively miniaturized by increasing its circumference and the length of current paths^14^. Consequently, the cell geometry may be modified according to Fig. 2 to decrease its size^15^. The transmission and reflection coefficients of the modified shape are drawn in Fig. 2a. The resonance frequency is decreased from 8.06 GHz to 5.9 GHz. The induced current density on the cell is shown in Fig. 2b. Observe that by meandering the loop, the current path increase, and the capacitance between the adjacent strips also increases, which leads to further miniaturization.

**Figure 3:**
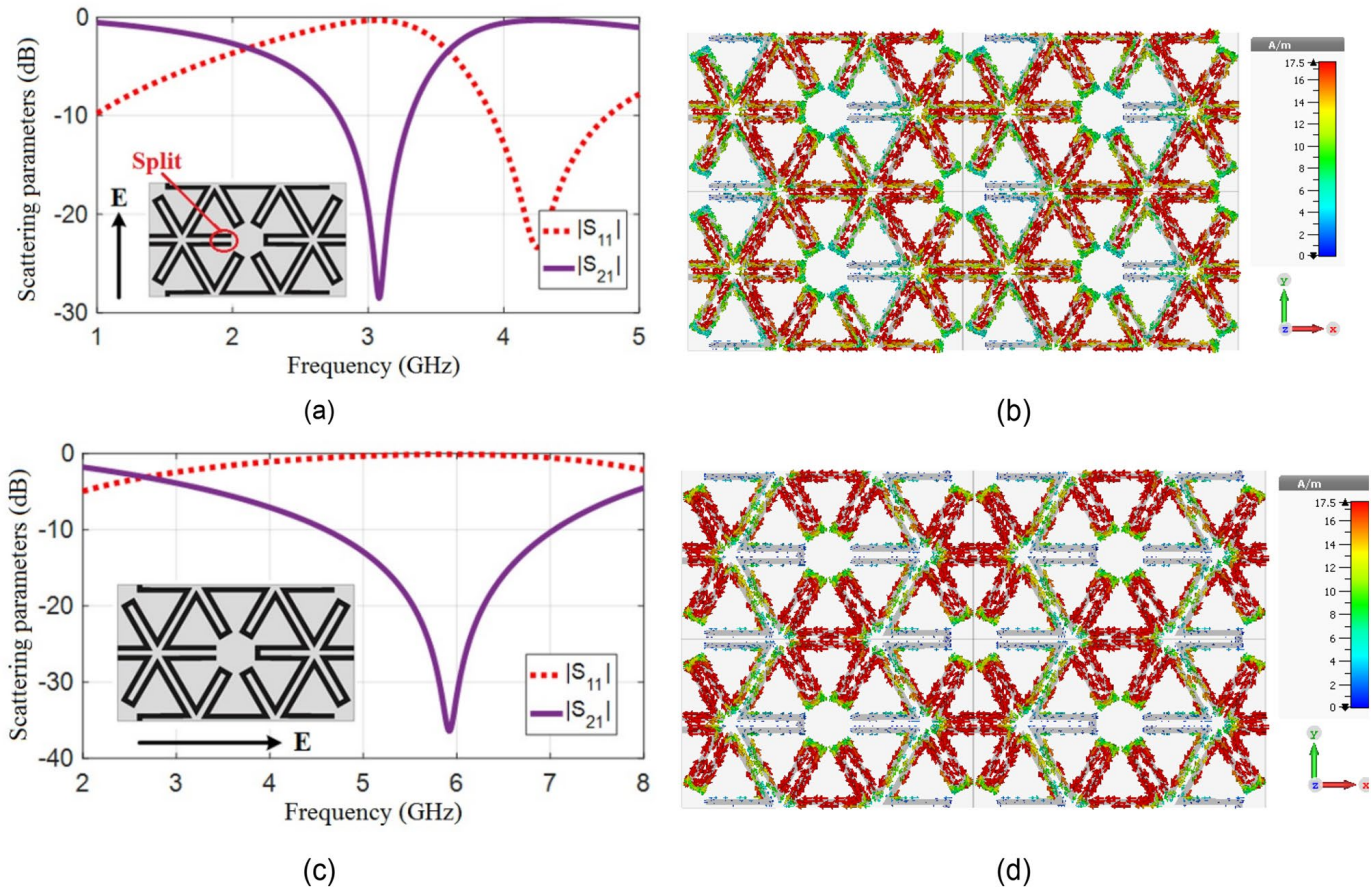
Simulation results of proposed split ring unit cell, (**a**) Scattering parameters for TE mode excitation. (**b**) Surface current at 3.1 GHz for TE mode excitation. (**c**) Scattering parameters for TM mode excitation. (**d**) Surface current at 5.9 GHz for TM mode excitation.

Implementation of a split in the ring (as in Fig. 3) causes the appearance of capacitance by the incident electric field E across the split. In this case, the resonance frequency decreased to 3 GHz and it became half (the current distribution of the split ring structure had one maximum). The performance of this structure resembles that of a half-wave dipole. The current distribution for the TE and TM modes at the related resonance frequencies are drawn in Fig. 3. In the case of TE mode, the E-field across the gap of split causes the capacitance to appear and the structure resonant at half a wavelength. In the case of TM mode, the E-field is parallel to the split and does not affect it. Therefore, the behavior of the structure is similar to a ring with a circumference equal to the wavelength.

**Figure 4:**
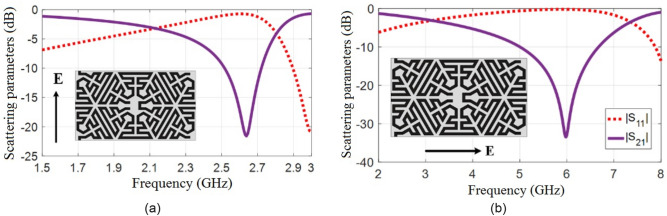
Simulation results of the meandered split ring unit cell. (**a**) TE mode, (**b**) TM mode.

For further miniaturization of the FSS structure, it may be meandered inside its available empty space. The proposed FSS configuration is shown in Fig. 4. Such meandering traces should be implemented by precision and limitation of fabrication, which is 0.2 mm for metallic trace width and spacing, the fabrication method is a laser plotter, exposure machine, developing machine, and acid machine that eventually limited by 0.2 mm.

The proposed unit cells are Polarization-sensitive FSS, and have many applications in wireless communication such as same-time spatial filters and reflectors in high-gain filtering antenna instead of the traditional polarizing grid^16^, metamaterial absorbers for application in polarization imaging, and polarization detecting^17^, polarization processing^18,19^, etc.

### Use of 2.5-D split rings for increasing the length of current paths

**Figure 5:**
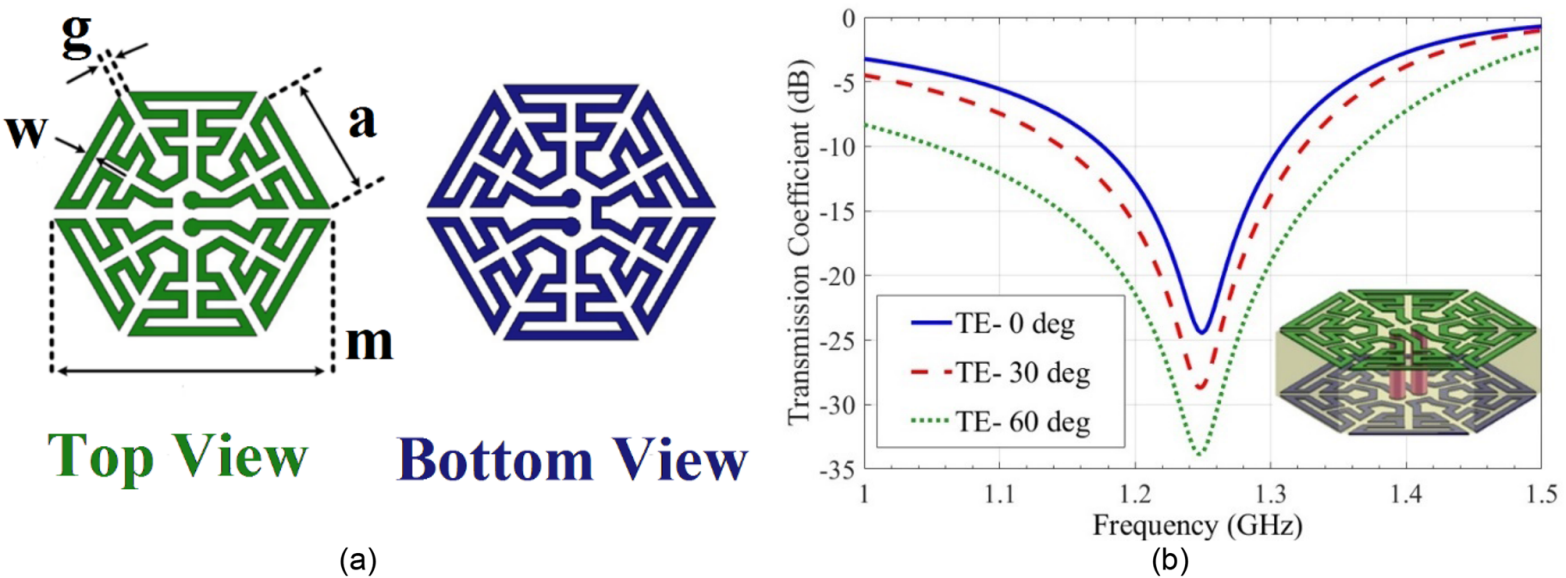
(**a**) Top and bottom layers of the proposed 2.5-D unit cell. (**b**) Simulation results of the unit cell for different incident angles.

An effective method for increasing the current path length and decreasing the resonance frequency is to use the two sides of the substrate for implementing the ring, in such a way that two parts of a ring are placed on its top and bottom sides. These two parts are connected by two vias, which provide current paths between them, the vias also add capacitance and inductances to the circuit diagram of FSS, which lead to its miniaturization and decrease of resonance frequency. Figure 5a shows the geometrical configuration of this single-cell FSS. It is made on substrate FR-4 (${\epsilon }_{r}=4.3$ and $\mathrm{tan}\delta =0.02$), $h=1.6\, mm.\, a=2.9 \,mm.\, g=0.2 \,mm. \,w=0.2 \,mm \,and \,m=6.3\, mm$. The transmission coefficient of proposed unit cell for TEz wave incidence at various angles are drawn in Fig. 5b.

Observe that this polarization has angular stability up to 60 to the ring operator due to its miniaturization by the proposed geometrical structure. Indeed, in an ultra-miniaturized unit cell, the dimension of the unit cell is very smaller than the wavelength of resonant frequency, and the variation of incident angles does not sense for a periodic structure. Also, angular stability also depends on unit cell geometry^20^. The transmission zero occurs at 1.25 GHz, which is relatively constant with respect to the angle of wave incidence.

Table 1 shows a comparison of the proposed FSS with those available in the literature. The FSS in Ref.^13^ is a multilayer structure with 46 vias and the FSS in Ref.^21^ uses 80 vias, which makes their fabrication quite complex, which leads to fabricated problems (the connection between trace and vias). Reference^22,23^ had 29 vias and 28 vias per unit cell respectively, and ultra miniaturization needed more than 20 vias but this paper, investigated split ring structure to derive ultra miniaturization.

**Table 1:** Comparison of the proposed FSS with other structures in the literature.

Ref	Period (mm)	${\epsilon }_{r}$	${f}_{r}$(GHz)	Element size	Angular stability	Structure's profile	Number of vias
^13^	3.5	4.4	2.47	$0.038{\lambda }_{0}\times 0.038{\lambda }_{0}$	45°	Multilayer 2.5-D	46
^15^	–	4.4	2.5	$0.081{\lambda }_{0}\times 0.081{\lambda }_{0}$	60°	1 layer	0
^21^	16	4.3	0.9	$0.048{\lambda }_{0}\times 0.048{\lambda }_{0}$	80°	2 layers cascaded 2.5-D	80
^22^	4.4	4.3	1.76	$0.026{\lambda }_{0}\times 0.026{\lambda }_{0}$	45°	1 layer 2.5-D TML-FSS	29
^23^	6.2	4.3	2.265	$0.033{\lambda }_{0}\times 0.033{\lambda }_{0}$	30°	1 layer 2.5-D	28
This work	6.3	4.3	1.25	$0.026{\lambda }_{0}\times 0.026{\lambda }_{0}$	60°	1 layer 2.5-D	2

### The equivalent circuit

**Figure 6:**
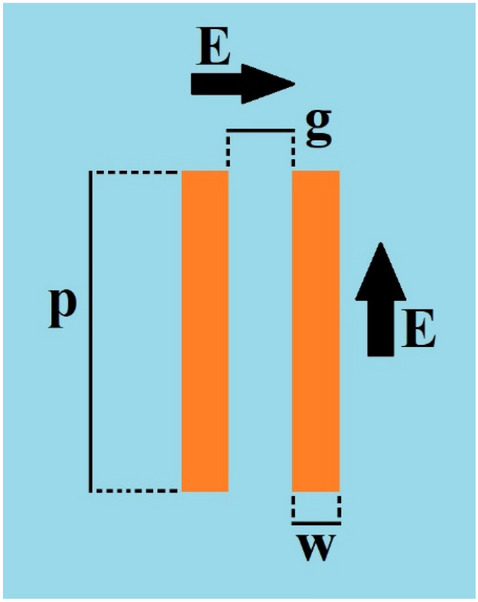
Parallel wires are used as the inductor or capacitor based on the electric field direction.

To investigate the physical behavior of a unit-cell geometry and its dimensions on its performance together with a comparison with the full-wave simulation of its responses, an equivalent circuit of the proposed FSS is obtained. Reference^24^ investigated the relation between capacitive and inductive elements with the geometry of structure and incident wave in strip line as shown in Fig. 6, where *L* is the strip inductance, *C* is the capacitance between the two adjacent patches, which is determined by the strip length *P*, the gap *g* between adjacent strips and the dielectric permittivity (*ε*), the strip width *w*, and the permeability *µ* of the structure.

1 $$C= {\varepsilon }_{0}{\varepsilon }_{r}\frac{2p}{\pi }cos\theta \left[lncosec\left(\frac{\pi g}{2p}\right)\right],$$

2 $$L= {\mu }_{0}{\mu }_{r}\frac{p}{2\pi }cos\theta \left[lncosec\left(\frac{\pi w}{2p}\right)\right].$$

**Figure 7:**
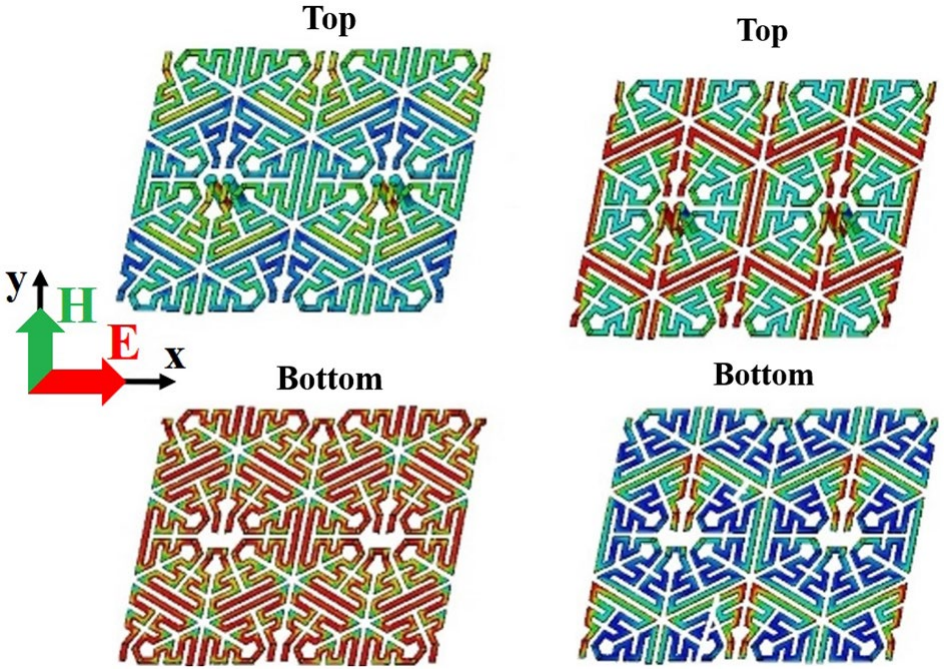
(**a**) Surface current. (**b**) Electric field.

Therefore, maximum current will be induced in a track parallel to the electric field and maximum electric field distribution will appear across two parallel tracks perpendicular to the incident electric field. Consequently, inductive effects will appear in tracks carrying induced currents and capacitive effects will appear in the regions with high electric field concentration (Fig. 7). In fact, by the analysis and variation of components in the equivalent circuit, the desired transmission coefficient can be determined and their limitations identified; then, by the variation of gaps between tracks and track widths and also with due consideration of fabrication limitations, the unit-cell may be modeled. Also, the designer can use equivalent circuit to reduce the design efforts and simulation tasks for a particular structure with the desired specifications. The equivalent circuits actually provide some primary design guidelines^25,26^.

The proposed unit cell is a split ring resonator and the current and electric field distribution for TE mode (electric field along the *x*-axis) is shown in Fig. 7, and the electric field of the TM polarization incident mode is along the positive *y*-axis. When the incident polarization is TE, the incident electric field is along the *x*-axis direction, which results in an even-mode current flowing along the stub and two anti-phase currents in the two arms, because the current distribution is maximum at the middle of the unit cell track and minimum at the first and end of unit cell track. On the other hand, under TM polarization, the incident electric field is along the *y*-axis direction, which excites two in-phase currents in the two arms and an odd-mode distribution along the stub, and the current distribution is minimum at the middle of the unit cell track. Regarding this point, the unit cell has equal potential spots on its track which is used for determining the equal circuit model^18,19^.

**Figure 8:**
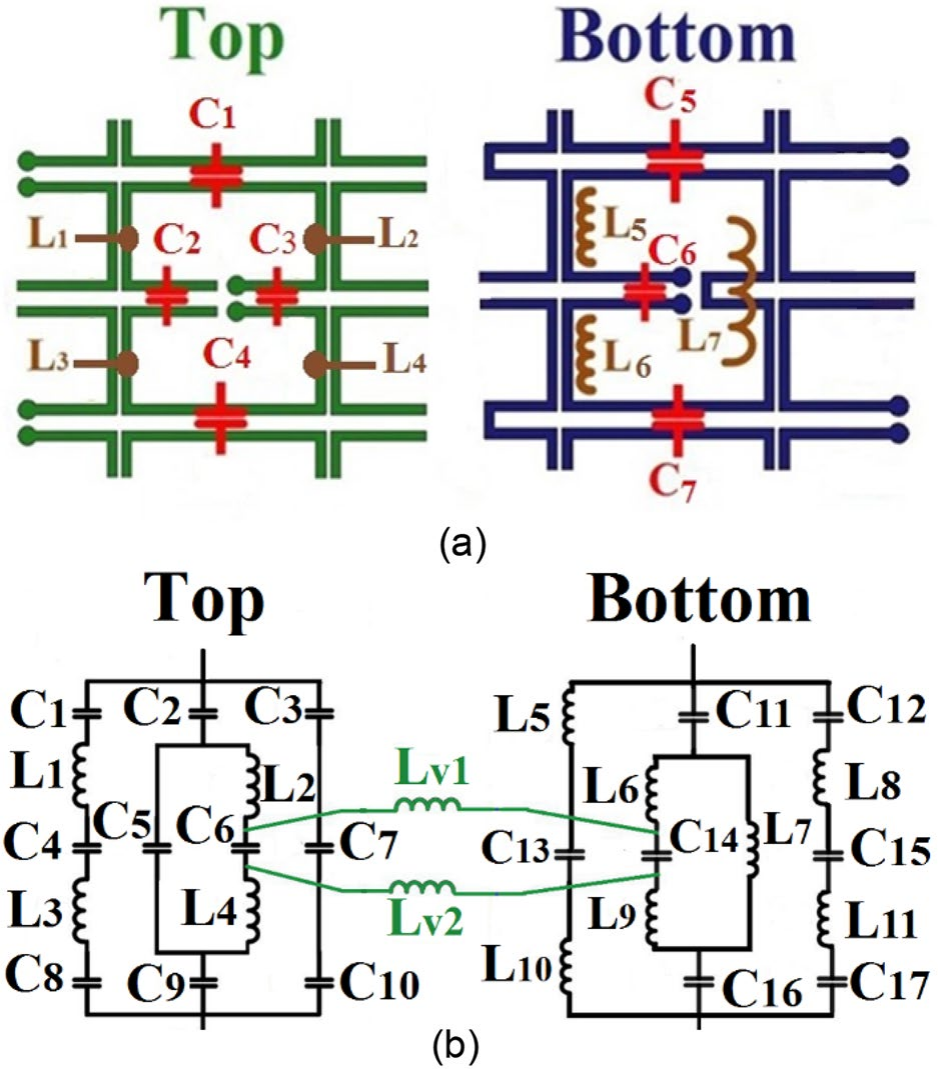
(**a**) The simplified ring at the adjacent cell. (**b**) Proposed unit cell equivalent circuit.

To depict the equivalent circuit's components of the hexagonal unit cell more obviously, Fig. 8 shows the simplified adjacent as square unit cells on both sides of the substrate. The equivalent capacitances and inductances are also indicated.

**Figure 9:**
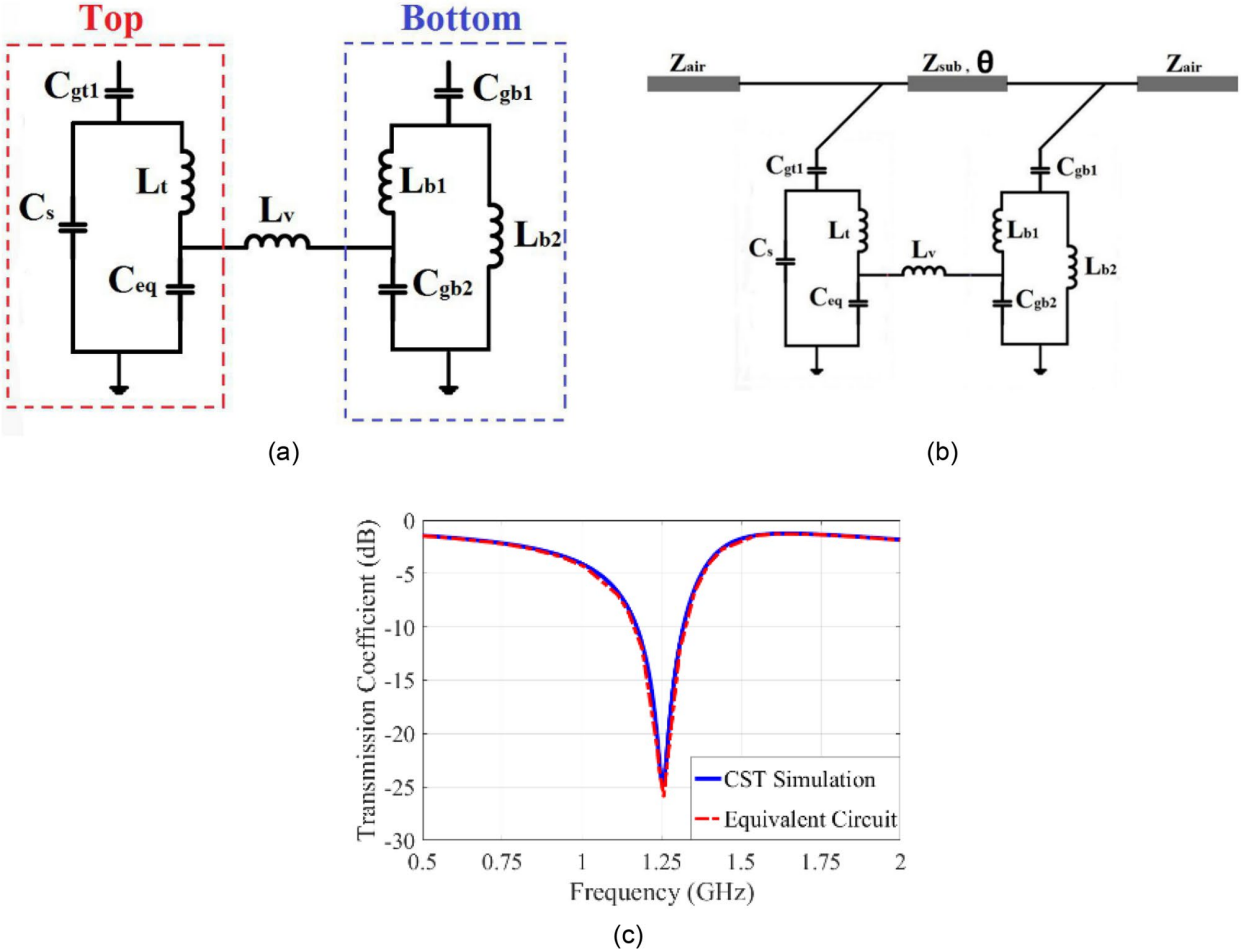
(**a**) The simplified equivalent circuit of structure. (**b**) The equivalent circuit of the proposed unit cell with the substrate. (**c**) Comparison of results from the simulation and equivalent circuit.

The inductances of two vias connecting the two sides are indicated as ${L}_{v1}$ and ${L}_{v2}$ (Fig. 8b). The effect of inductors ${L}_{1}$, ${L}_{2}$ can be ignored due to the negligible current distribution at these locations on the unit cell. Also due to unit cell geometry, the capacitors ${C}_{1}={C}_{4}$, ${C}_{5}={C}_{7}$, and inductors ${L}_{2}={L}_{4}$ and ${L}_{5}={L}_{6}$. Therefore, these points are equipotential. Also, since a half-wave current fits on the unit cell, ${180}^{\circ }$ phase difference falls at the split section of the ring and ${90}^{\circ }$ phase difference appears at the lower section of the unit cell between vias. Due to the ${180}^{\circ }$ phase difference between the ${C}_{2}$ terminals, it can be divided into two equal series capacitors. Also, the interconnection between the two new capacitors can be grounded. Also, the same deduction is valid for ${C}_{6}$. Accordingly, the equivalent circuits of the two sides of the proposed FSS may be arrived at as shown in Fig. 9a. The values of elements of the equivalent circuit may be evaluated from the equivalent circuit in Fig. 9b.

The free space on the two sides of the unit cell may be modeled as a transmission line of characteristic impedance ${Z}_{air}= 377\Omega$ and the characteristic impedance of substrate is ${Z}_{sub}={Z}_{air}/\surd {\varepsilon }_{r} =180\Omega$ for FR-4. The properties of substrate such as height (h), dielectric consist (*ε*_*r*_), and loss tangent (*tanδ*) affect both the amplitude and phase of transmission coefficient of FSS cells as $\theta =\beta l$^12^. Figure 9c shows comparison of results from the simulation and equivalent circuit. The values of elements of proposed equivalent circuit are given in Table 2.

**Table 2:** The values of circuit elements in Fig. 9a.

Element	Value	Element	Value	Element	Value
${C}_{gt1}$	0.38 pF	${L}_{b1}$	5.64 nH	${C}_{eq}$	0.17 pF
${L}_{t}$	1.78 nH	${L}_{b2}$	7.30 nH	${C}_{gb1}$	0.07 pF
${L}_{v}$	0.91 nH	${C}_{s}$	0.52 pF	${C}_{gb2}$	0.13 pF

The frequency responses of the proposed equivalent circuit achieved by the ADS^27^ simulation software and the CST full-wave software^28^ coincide very well, which indicates that the proposed equivalent circuit gives a real physical model and interpretation of the proposed FSS structure as shown in Fig. 9c.

**Figure 10:**
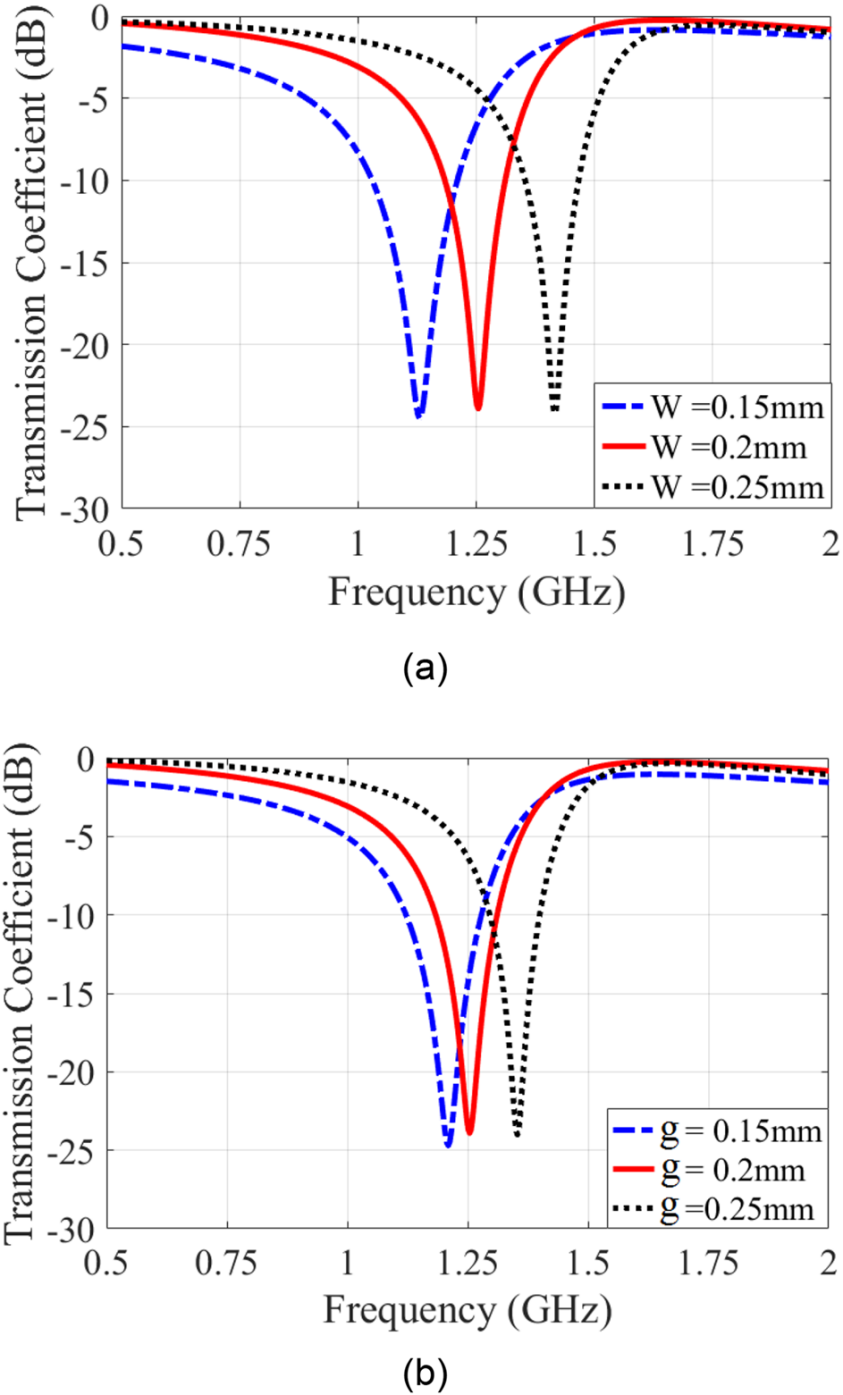
Transmission coefficient for various values of: (**a**) *w*. (**b**). *g*.

Figure 10 shows the frequency response of the transmission coefficient for various values of *w* and* g*. The variations of resonance frequency and bandwidth of the frequency response of FSS may be obtained from its equivalent circuit. It is observed that the frequency corresponding to the resonance frequency depends on the size of the inductors more specifically, the resonance frequency decreases by increasing the size of the inductors (decreasing the *w* values). These results are verified by the full-wave software, by changing the width of line tracks (*w*), as shown in Fig. 10a. In this case, the bandwidth is increased by decreasing the '*w*', due to inductors increasing, and it may obtain by the proposed equivalent circuit. The parameters that affect the size of capacitors appearing in the unit cell are the spacing of tracks and the properties of substrate according to the relation C = εrε0(A/d)^24^. Therefore, the resonance frequency may be controlled. The variation effect of the track spacing '*g*' on the FSS transmission response is shown in Fig. 10b. Observe in Fig. 10b that by decreasing the gap between track (*g*) the resonance frequency decreases and the bandwidth increases respectively. The aforementioned results indicate how the bandwidth may be increased by increasing the capacitance, which amounts to decreasing spacing (*g*), and by increasing the inductance through decreasing track widths, and all of these concepts can be studied by equivalent circuit models in ADS software.

### Fabrication and measurement

**Figure 11:**
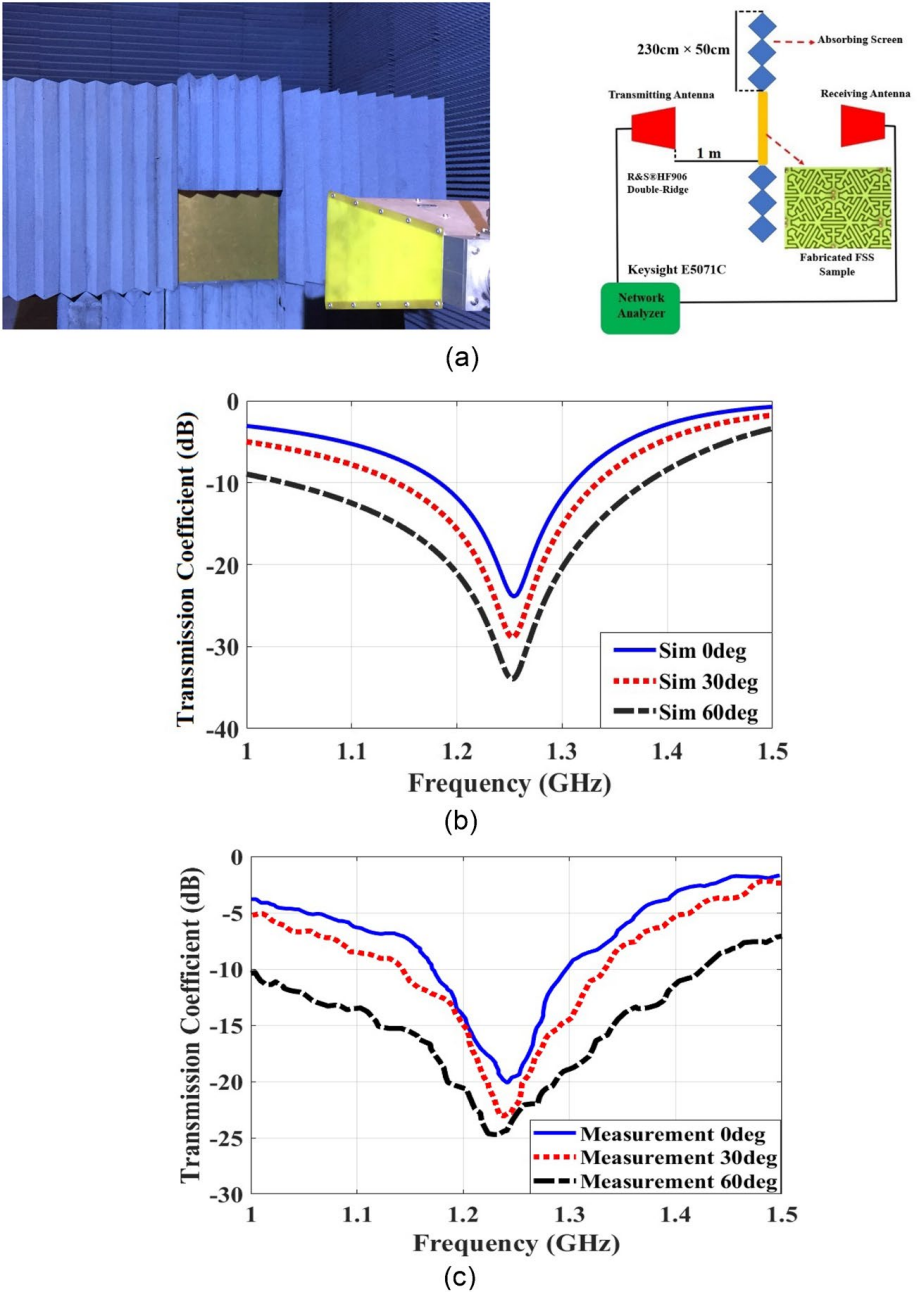
(**a**) The measurement set-up in the anechoic chamber. (**b**) The transmission coefficient simulations. (**c**) The transmission coefficient measurements.

A prototype model of the proposed FSS is designed, fabricated, and tested. The dimensions of FSS are 350mm × 350mm. The measurement frequency range is 1–1.5 GH equal to 20–30 cm for free space wavelength. In the case of usage of FR-4 substrate with ${\varepsilon }_{r}$= 4.3 and loss tangent = 0.02, the effective wavelength is equal to 12.27–18.4 cm respectively due to $\lambda ={\lambda }_{0}/(\sqrt{(1+{\varepsilon }_{r})/2}$). The same antennas with 54-degree 3-dB bandwidth and less than 35 cm far field range in 1–1.5 GHz is used for transmit and receive antennas (The standard R&S®HF906 Double-Ridge Antenna is used), and the model of Network Analyzer is Keysight E5071C. Since its’ 3 dB beam-width is about 54°, it covers an area in the far field. In order to direct the wave solely through the FSS window area, absorber blocks are located around it. For the calibration, the transmission coefficient (S21) is first measured. It is measured again for the case of locating the FSS between the two antennas. The difference between the two measurements gives the transmission coefficients of the FSS structure. In order for the measurements to be valid, the FSS should be positioned in the far fields of the transmitter and receiver antennas, whereby its transmission response is obtained. For the measurements of the transmission coefficients at various angles, the plane of FSS should be rotated about its central axis by appropriate angles and the signals of the receiver antenna be recorded. The measurement setup and results of transmission coefficients at the incident angles of 0, 30, and 60 degrees are shown in Fig. 11. The measured FSS transmission coefficients agree well with the computer simulation values. For valid measurement results, the FSS structure should be positioned in the far-field regions of the transmitter and receiver antennas. Due to the fabrication process error on track width, the transmission zero has shifted downwards, which can explain by the EC model.

## Conclusion

In this paper, a miniaturized hexagonal meandered split ring FSS is proposed using a 2.5-D closed loop that is compared to similar FSS structures in the literature. The advantages of this structure are miniaturization and polarization dependence which are used in detecting and sensing applications. Its equivalent circuit has been derived and the frequency response of it agrees very well with full wave simulation. Consequently, the variation of geometry parameters' effect on frequency response is investigated and can explain with an equivalent circuit model. A prototype model of the proposed FSS is designed, fabricated, and measured as proof of concept.

## Data Availability

The data acquired during the current study are not publicly available due to privacy/ethical restrictions but are available from the corresponding author on reasonable request.
